# Bacterial isolation and antibiotic susceptibility from diabetic foot ulcers in Kenya using microbiological tests and comparison with RT-PCR in detection of *S. aureus* and MRSA

**DOI:** 10.1186/s13104-019-4278-0

**Published:** 2019-04-29

**Authors:** Daniel M. Mutonga, Marianne W. Mureithi, Nancy N. Ngugi, Fredrick C. F. Otieno

**Affiliations:** 1 Institute of Tropical and Infectious Diseases, College of Health Sciences, University of Nairobi, P.O Box 19676-00202, Nairobi, Kenya; 2 KAVI-Institute of Clinical Research, P.O Box 19676-00202, Nairobi, Kenya; 3 Department of Medical Microbiology, College of Health Sciences, University of Nairobi, P.O Box 19676-00202, Nairobi, Kenya; 4 Department of Medicine, Kenyatta National Hospital, P.O. Box 20723-00202 Nairobi, Kenya; 5 Department of Clinical Medicine and Therapeutics, College of Health Sciences, University of Nairobi, P.O Box 19676-00202, Nairobi, Kenya; 6 P.O Box 11692-00100, Nairobi, Kenya

**Keywords:** Diabetic foot ulcers, Kenya, Multi-drug resistant organisms, Methicillin-resistant *S. aureus*, Polymerase chain reaction

## Abstract

**Objectives:**

Diabetic foot ulcers (DFUs) often lead to hospital admissions, amputations and deaths; however, there is no up-to-date information on microbial isolates from DFUs and no mention of utilization of molecular techniques in Sub-Saharan Africa. We conducted a cross-sectional study among 83 adult patients at a tertiary hospital in Kenya over 12 months. The study aimed to isolate, identify bacteria, their antibiotic susceptibility patterns in active DFUs, and to compare standard microbiological methods versus a real-time PCR commercial kit in the detection of *Staphylococcus aureus* DNA and methicillin-resistant *S. aureus* (MRSA) DNA.

**Results:**

Eighty swabs (94%) were culture-positive; 29% were Gram-positive and 65% were Gram-negative. The main organisms isolated were *S. aureus* (16%), *Escherichia coli* (15%), *Proteus mirabilis* (11%), *Klebsiella pneumoniae* (7%) and *Pseudomonas aeruginosa* (7%). The bacterial isolates showed resistance to commonly used antibiotics such as ampicillin, amoxicillin, cefepime, ceftazidime, cefuroxime, clindamycin, erythromycin, piperacillin–tazobactam, tetracycline and trimethoprim–sulphamethoxazole (TMPSMX). Thirty-one percent of the *S. aureus* isolated and 40% of the Gram-negatives were multi-drug resistant organisms (MDROs). There was a high prevalence of nosocomial bacteria. MRSA were not identified using culture methods but were identified using PCR. PCR was more sensitive but less specific than culture-based methods to identify *S. aureus.*

**Electronic supplementary material:**

The online version of this article (10.1186/s13104-019-4278-0) contains supplementary material, which is available to authorized users.

## Introduction

It is estimated that 10–15% of diabetic patients will develop diabetic foot ulcers (DFUs) at some point in their life [1, 2]. In Africa, the overall prevalence of DFUs was found to be 13% in a recent meta-analysis [3]. At presentation, about half of DFUs are clinically infected [4]. *Staphylococcus aureus* and beta-haemolytic *Streptococci* are the most common causes of skin infections [5–11]. In resource-poor countries however, Gram-negatives like *Pseudomonas aeruginosa* are more prevalent [6, 10]. In Kenya, *S. aureus* and *Escherichia coli* were found to be the most common organisms in DFIs [12]. More recently, 73.2% of DFUs were infected while 26.8% were culture-negative [13]. Fungal infections may also cause DFIs [14].

Antimicrobial resistance (AMR) is an emerging problem globally. Methicillin-resistant *S. aureus* (MRSA) was first observed in the early 1960s and has been associated with increased hospital stay, healthcare costs and mortality [15]. MRSA represented 4.7% of *S. aureus* isolated in a study in Morocco [16]. In Brazil, 33% cases of MRSA (cefoxitin-resistant) were vancomycin-resistant [17]. Multi-drug resistant organisms (MDROs) are bacteria that are resistant to more than one or more classes of antibiotics. In Tanzania, antibiotic susceptibility tests (AST) of bacterial isolates from DFUs revealed a high AMR [18].

Polymerase chain reaction (PCR) is a molecular method that can be used to identify bacterial species by amplifying the 16S ribosomal RNA (rRNA) gene [4, 10]. Real-time PCR (RT-PCR) allows detection of DNA or RNA through production of fluorescence light during the reaction. In Sub-Saharan Africa, there is a lack of up-to-date information on microbial isolates from diabetic foot ulcers and no mention of utilization of molecular techniques. The only available study from Africa is from Algeria where sequencing target genes identified a high prevalence of Gram-negative bacilli (54.9%) and MDROs (58.5%) [19].

The objective of this present study was therefore to isolate bacteria and determine their antibiotic susceptibility patterns in patients with infected DFUs using culture-based methods and to compare the differences between microbiological methods and RT-PCR in detecting *S. aureus* and MRSA in a sub-Saharan setting, which is facing an escalating AMR with extensive health, economic and societal implications.

## Main text

### Methods

#### Study design and subjects

This cross-sectional study was conducted at the Kenyatta National Hospital (KNH), Nairobi, Kenya—a national referral and teaching hospital. Eighty-three adult diabetic patients with any type of diabetes and having active foot ulcers were recruited by consecutive sampling from September 2017 to August 2018. Active foot ulcers were defined as non-healed ulcers during physical examination and were thought to be more likely infected.

#### Microbiological methods

After rinsing the wound area with normal saline, samples were collected using sterile cotton swabs from the centre of the diabetic wound and taken to the KNH Microbiology Laboratory immediately or within 2 h. On Day 1, specimens were inoculated using the streak method on Sheep Blood Agar and CLED Media and incubated under aerobic conditions at 35–37 °C for 24–48 h. On Day 2, growth was noted as colonies on the culture media and the most predominant colony isolated using standard microbiological and biochemical tests. The VITEK^®^ 2 machine (bioMe´rieux, Durham, United States) was then utilized for further identification and AST.

#### Screening for *S. aureus* and MRSA DNA using RT-PCR

All specimens were stored at − 20 °C to − 80 °C for subsequent DNA isolation and PCR analysis at Biozeq Kenya Molecular Laboratory (based at KAVI-Institute of Clinical Research, University of Nairobi). Fifty-one samples were randomly selected from the 83 recruited patients and dissolved in 200 µL to 500 µL of Dulbecco’s phosphate buffered solution (Sigma^®^-Aldrich, Steinheim, Germany). Automated DNA extraction was performed using QIASymphony Kit (Qiagen^®^, Hilden, Germany) according to manufacturer’s instructions. PCR-amplification and real-time hybridization were conducted using the MRSA Quant Real-TM kit (Sacace™ Biotechnologies, Como, Italy). Amplification was set up in a 1.0 µL PCR tubes containing 15 µL of PCR Master Mix (PCR mix-1 FRT MRSA, PCR-mix-2 FRT, TaqF polymerase, and Internal Control). The reaction tubes were subjected to Thermal cycling reactions on a Rotor-Gene Q machine (Qiagen, Hilden, Germany) comprising of 15 min at 95 °C followed by 5 cycles of 15 s at 95 °C, 30 s at 60 °C, and 15 s at 72 °C, and finally 40 cycles of 15 s at 95 °C, 30 s at 55 °C and 15 s at 72 °C for measuring fluorescent signal.

#### Quality control

Positive controls for microbiological tests were recently collected (< 7 days) positive specimens of bacteria from human samples that were stored at room temperature in cotton swabs in a safety cabinet. For the RT-PCR tests, quality control was assured by running 4 additional samples alongside the 51 specimen: Positive control, Negative control, DNA Quality Standard (QS) 1 MRSA and DNA QS2 MRSA.

#### Statistical analyses

Microsoft Excel was used for data entry and data analysis. Data was represented as frequencies, percentages, tables and charts. Comparison of microbiological and RT-PCR was based on absolute numbers, sensitivity, specificity, positive predictive value (PPV), and negative predictive value (NPV). Both culture and real-time PCR were considered as the gold standard.

### Results

#### Bacterial isolates and antimicrobial sensitivity tests

The most predominant growth on the culture plate per specimen was isolated. Out of 85 culture and susceptibility tests performed from 83 patients, 78 swabs had mono-microbial growth, 1 had poly-microbial growth (2 isolates) and 5 had no growth. Most organisms (64.71%, n = 55) were Gram-negative and (29.41%, n = 25) organisms were Gram-positive. The most common organisms isolated were *S. aureus* (16.47%, n = 14), *E. coli* (15.29%, n = 13), *Proteus mirabilis* (10.59%, n = 9), *Klebsiella pneumoniae* (7.06%, n = 6) and *P. aeruginosa* (7.06%, n = 6) (see Additional file 1: Figure S1). Some of the other rare bacteria isolated from patients included *Staphylococcus lentus*, *Staphylococcus simulans*, *Staphylococcus xylosus*, *Acinetobacter baumannii*, *Burkholderia cepacia*, *Kocuria kristinae*, *Leuconostoc mesenteroides*, *Pantoea agglomerans*, *Providencia stuartii* and *Raoultella ornithinolytica*.

**Fig. 1:**
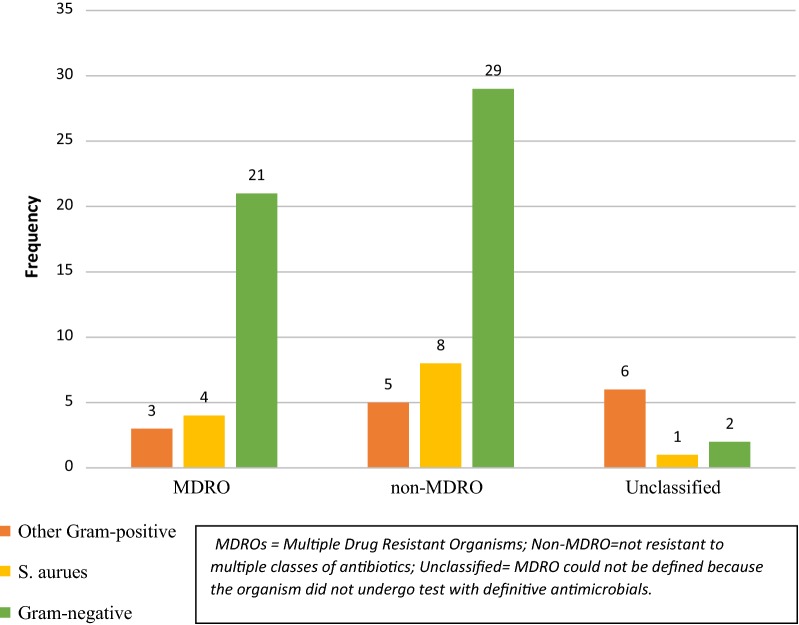
Distribution of MDROs among Gram-positive and Gram-negative organisms. The chart in this figure illustrates the distribution of MDROs among Gram-positive and Gram-negative bacteria. *S. aureus* and other Gram-positive organisms are displayed on different bars. Antibiotic susceptibility was not determined for tests that showed no growth, *Kocuria kristinae*, and *Leuconostoc mesenteroides.* AST was also not performed on a sample suspected to have contaminants but was positive for *S. aureus* on RT-PCR; therefore n = 13

*Staphylococcus aureus* was highly resistant to benzylpenicillin and trimethoprim–sulphamethoxazole (TMPSMX) but sensitive to cefoxitin, oxacillin, nitrofurantoin, levofloxacin, linezolid, and vancomycin. There was no MRSA identified using microbiology tests. Isolates of *S. epidermidis*, *S. intermedius* and *S. simulans* were either 100% resistant, 50% resistant or 100% sensitive to the antibiotics tested (Additional file 2: Table S1). *E. coli* was highly resistant to ampicillin, aztreonam, cefuroxime and TMPSMX but sensitive to amikacin and nitrofurantoin (refer to Additional file 2: Table S2). *P. mirabilis* showed a similar resistance to ampicillin but sensitivity to amikacin. *P. aeruginosa* was sensitive to ampicillin, amoxicillin–clavulanic acid, aztreonam, ceftazidime, ciprofloxacin, nitrofurantoin and TMPSMX. *S. fonticola* species showed resistance to ampicillin, amoxicillin, cefazolin, cefepime, ceftazidime, piperacillin-tazobactam and TMPSMX. About a third (30.77%, n = 4) of the *S. aureus* were MDROs while 40.38% (n = 21) Gram-negative bacilli were MDROs (Fig. 1).

#### Culture-versus-molecular tests

**Fig. 2:**
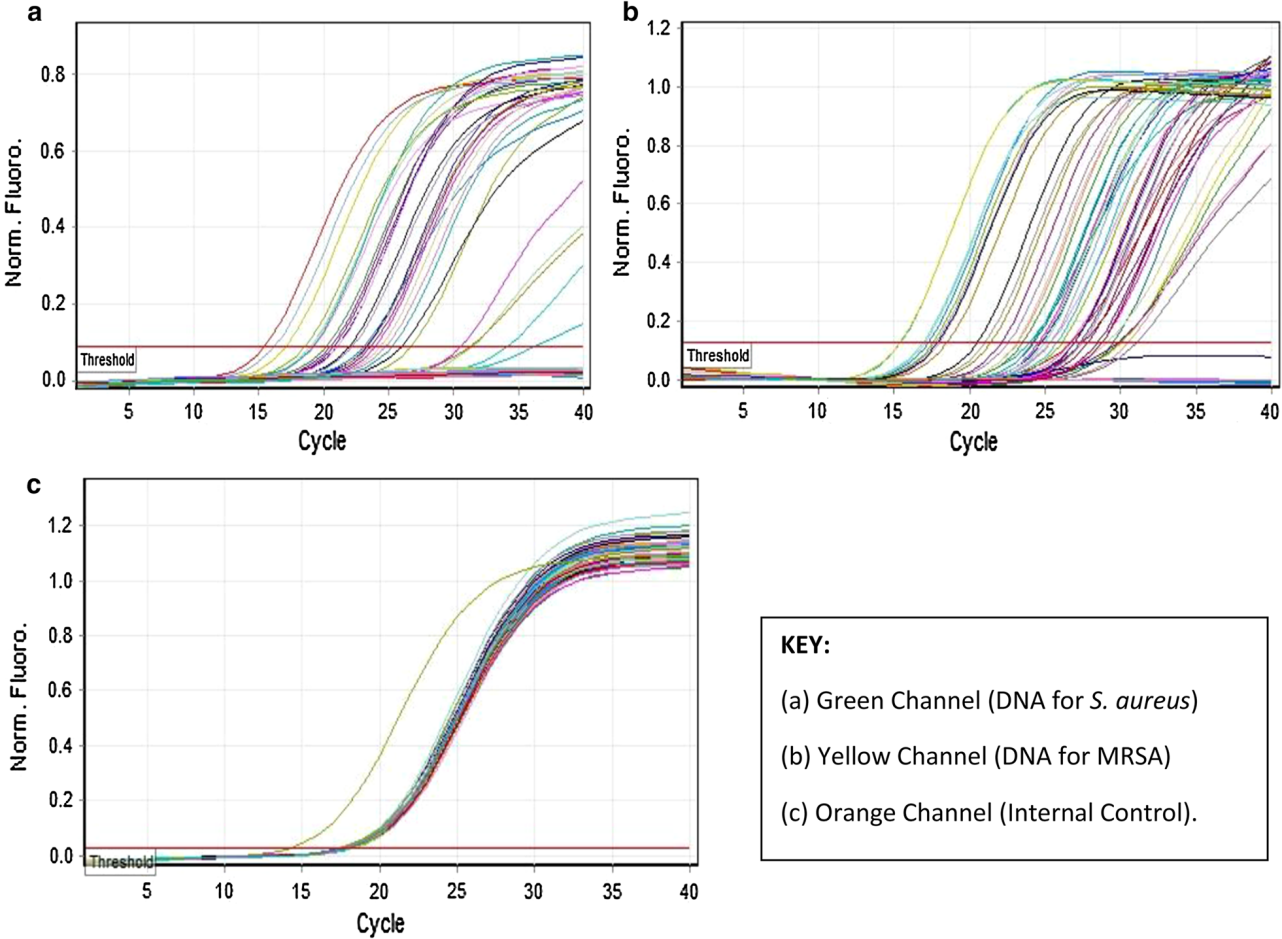
Quantitation data for Cycling A (PCR Reports). As shown in this figure, the C_t_ used was ≥ 20 since the exponential of the sigmoid curve of the Internal Control (Orange Channel) begins at this point

Molecular tests were performed on 51 out of the total 85 samples tested (Fig. 2). The RT-PCR results of the 51 samples were compared with the respective culture results for the detection of *S. aureus* and MRSA. For the Gram-positive pathogens, 11 were culture-positive for *S. aureus* while 7 yielded other *Staphylococcus* sp. RT-PCR for *S. aureus* was positive for 9 out of the 11 culture-positive results (see Additional file 2: Table S3). Five samples positive for *S. aureus* but negative for MRSA on culture-based methods were positive for MRSA on RT-PCR. Two samples positive for other *Staphylococcus* sp. and negative for *S. aureus* on culture were positive for MRSA on RT-PCR. One sample with other *Staphylococcus* sp. suspected to be skin contaminants based on microbiological tests underwent RT-PCR. The sample was negative for *S. aureus* on culture tests but positive for *S. aureus* and MRSA DNA on RT-PCR.

One sample without any growth was subjected to RT-PCR. The sample was positive for *S. aureus* DNA but not MRSA DNA. Four (12.5%) samples that had Gram-negative bacteria based on culture tests were also positive for *S. aureus* on RT-PCR while 6 (18.8%) samples from these batch were positive for MRSA DNA (Additional file 2: Table S4). Of importance is that MRSA were not identified using culture methods but were identified using PCR. There was amplification for MRSA in one sample that was culture positive for *S. aureus* but negative during RT-PCR for this pathogen (Additional file 2: Table S3). Atypical amplification for MRSA occurred in four samples that were both culture and RT-PCR negative for *S. aureus* (Additional file 2: Table S4).

Further comparison was then made by statistical analyses. Firstly, culture methods were considered the assay and RT-PCR as the reference. The sensitivity of the VITEK^®^ 2 machine to detect *S. aureus* was 90.9% while the specificity was 82.5% (Table 1 and Additional file 2: Table S5). The PPV was 58.8% while the NPV was 97.0%. The sensitivity and PPV of the culture tests to detect MRSA could not be calculated due to missing culture-positive results (Additional file 2: Table S5). However, its specificity was 72.7% and NPV was 100%. In the second case, the RT-PCR was considered the assay and culture tests as the reference. The sensitivity, specificity, PPV and NPV of the RT-PCR to detect *S. aureus* were 58.8%, 97.0%, 90.9% and 66.7% respectively (Table 1). For detection of MRSA, the specificity for the RT-PCR was 100% while the NPV was 72.7%.

**Table 1:** Comparison of RT-PCR and culture-based methods of *S. aureus* and MRSA in DFUs

Species	Reference	Assay	Sensitivity (%)	Specificity (%)	PPV (%)	NPV (%)
*S. aureus*	Culture	RT-PCR	90.9	82.5	58.8	97
	RT-PCR	Culture	58.8	97	90.9	66.7
MRSA	Culture	RT-PCR		72.7		100
	RT-PCR	Culture		100		72.7

In this Table, both culture-methods and RT-PCR were used as gold standards. In the column heading, the gold standard is the “Reference” while the method being tested, the “Assay”

### Discussion

DFU is a chronic issue that contributes significantly to morbidity and mortality. In this study, over 90% of the DFUs were infected. This was higher than in earlier studies in Kenya, Tanzania, and Libya where approximately 70% of DFUs had positive cultures [13, 18, 20]. *S. aureus* was the most predominant single species isolated in DFUs as reported in other studies [2, 6–10, 12, 17, 18, 20]. In this current study, Gram-negative bacteria were more predominant than Gram-positive organisms similar to studies in Morocco and Brazil [16, 17]. Similar to previous studies, *E. coli* and *P. aeruginosa* were common in this study [6, 10, 12, 17, 20].

In this study, there was high AMR among the Gram-negative organisms compared to the Gram-positive bacteria. In fact, MDROs mainly consisted of Gram-negative bacteria. *S. aureus* was sensitive to most antibiotics including vancomycin whereas no MRSA was identified by culture methods (cefoxitin screen). From previous studies, MRSA is predominant in DFUs and shows limited AMR [21, 22]. In Brazil, 22% of DFUs had MRSA following cefoxitin screen, and 33% of these were also resistant to vancomycin [17]. *S. aureus* and *E. coli* isolated from DFUs were classified as MDROs in this earlier study. From previous studies, antibiotics that used to work before are now showing increasing resistance [12, 18].

Biofilms, present in chronic wounds, are a defensive mechanism for bacteria against the effects of antibiotics and can explain the rise in AMR [10, 11]. Unjustified use of antibiotics is another cause of AMR, misuse of health resources and a burden to patients and their families [6, 23, 24]. From this present study, amikacin is effective against most Gram-negative bacteria. The high AMR to ampicillin should warrant care during empirical treatment of DFUs in this setting [25]. Further, although some *E. coli* isolates were resistant to meropenem (a third-line antibiotic); all were sensitive to nitrofurantoin (a first-line antibiotic). There is therefore need to use antibiotics judiciously and be guided by routine culture and susceptibility tests.

However, more accurate tests should be explored since culture-based methods have been reported to have a high number of false-negatives [8]. In the present study, molecular tests were more sensitive but less specific than culture-based methods. PCR revealed pathogens that had not been recognized by culture-methods such as MRSA species. Previous research reveals a higher specificity for culture tests when compared to RT-PCR as a reference while a lower sensitivity, a slightly higher PPV and a higher NPV [8]. Similar to this earlier study, RT-PCR revealed more *S. aureus* than identified through culture-methods [8]. PCR is therefore an effective way of species identification in patients with DFUs.

## Limitations

- Due to limited funding, only the most predominant organism was isolated from the samples. Further, anaerobic bacteria were also not identified.
- PCR technology may amplify dormant or dead bacteria in a sample.
- The study fails to explain the atypical amplification why RT-PCR is positive for MRSA but negative for *S. aureus*. Contamination or cross-reactivity is not a possibility since the RT-PCR kit should detect both *S. aureus* DNA and mecA gene specific for MRSA.

## Additional files

**Additional file 1: Figure S1.** Distribution of Gram-positive and Gram-negative bacteria isolated.

**Additional file 2: Table S1.** Resistance patterns for Gram-positive organisms; **Table S2.** Resistance patterns for Gram-negative organisms; **Table S3.** Comparison of microbiological and molecular tests for Gram-positive bacteria; **Table S4.** Comparison of microbiological and molecular tests for Gram-negative bacteria; **Table S5.** Distribution of organisms based on culture and RT-PCR results for *S. aureus* and MRSA.

## Abbreviations

AMR: antimicrobial resistance
AST: antibiotic susceptibility tests
DFUs: diabetic foot ulcers
KNH: Kenyatta National Hospital
MDROs: multi-drug resistant organisms
MRSA: methicillin-resistant *S. Aureus*
NPV: negative predictive value
PCR: polymerase chain reaction
PPV: positive predictive value
RT-PCR: real-time PCR
TMPSMX: trimethoprim–sulphamethoxazole
