# Supplementary figures and images for: Bacterial isolation and antibiotic susceptibility from diabetic foot ulcers in Kenya using microbiological tests and comparison with RT-PCR in detection of S. aureus and MRSA
Source: BMC Res Notes. 2019 Apr 29;12:244. doi: 10.1186/s13104-019-4278-0 (PMC6489269; doi:10.1186/s13104-019-4278-0)

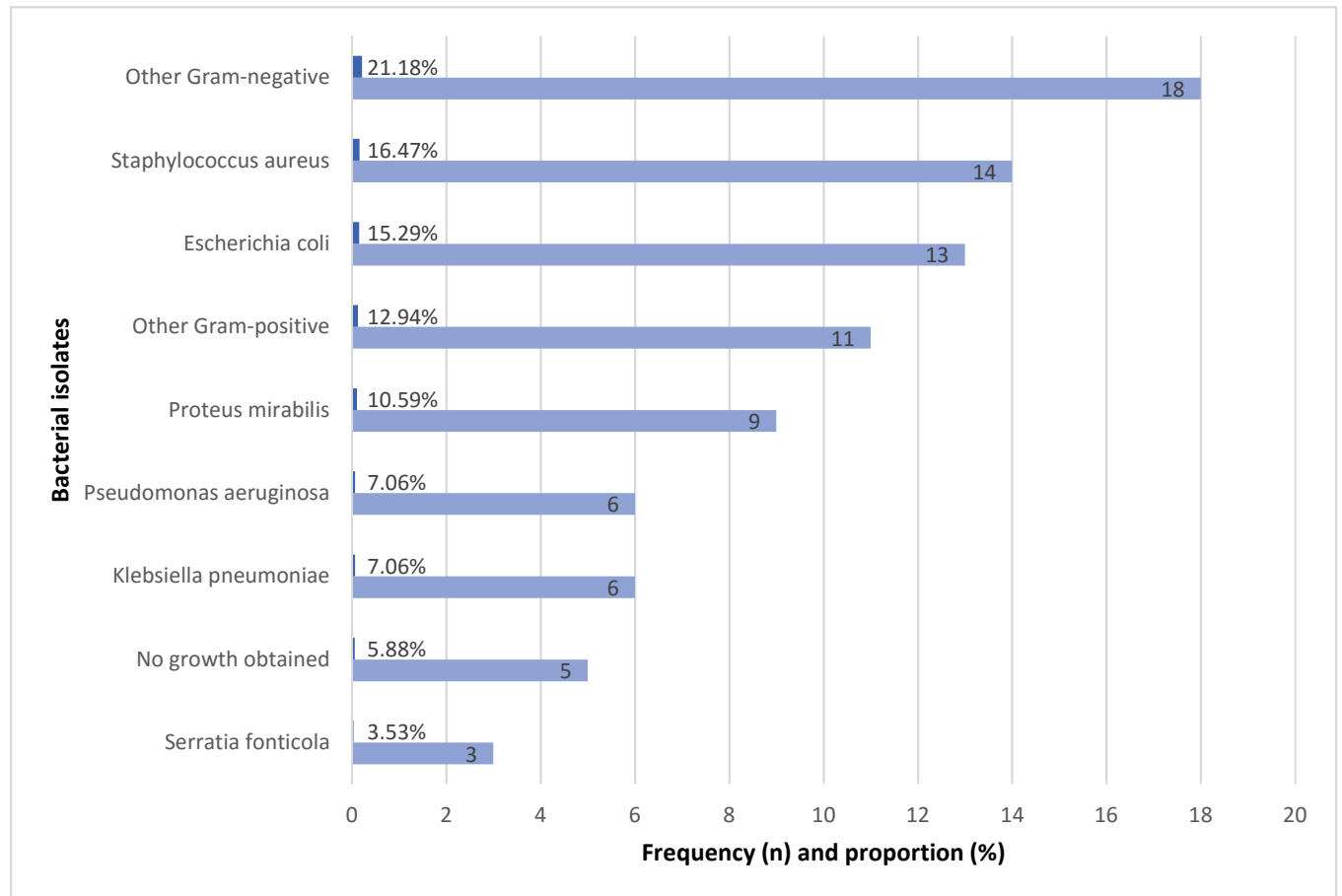

Supplement: Supplementary file 1 — Additional file 1: Figure S1. Distribution of Gram-positive and Gram-negative bacteria isolated. [file 13104_2019_4278_MOESM1_ESM.pdf]
