# Supplementary material for: Bacterial isolation and antibiotic susceptibility from diabetic foot ulcers in Kenya using microbiological tests and comparison with RT-PCR in detection of S. aureus and MRSA
Source: BMC Res Notes. 2019 Apr 29;12:244. doi: 10.1186/s13104-019-4278-0 (PMC6489269; doi:10.1186/s13104-019-4278-0)
Supplement: Supplementary file 2 — Additional file 2: Table S1. Resistance patterns for Gram-positive organisms; Table S2. Resistance patterns for Gram-negative organisms; Table S3. Comparison of microbiological and molecular tests for Gram-positive bacteria; Table S4. Comparison of microbiological and molecular tests for Gram-negative bacteria; Table S5. Distribution of organisms based on culture and RT-PCR results for S. aureus and MRSA. [file 13104_2019_4278_MOESM2_ESM.docx]

***Table S1: Resistance patterns for Gram-positive organisms***

|  | *n (%)* | | | |
| --- | --- | --- | --- | --- |
|  | *Staphylococcus aureus*  *n=14* | *Staphylococcus epidermidis*  *n=2* | *Staphylococcus intermedius*  *n=2* | *Staphylococcus simulans*  *n=1* |
| Benzylpenicillin | 12 (85.7) | 2 (100.0) | 2 (100.0) | 1 (100.0) |
| Beta Lactamase | 0 (0.0) | ND | 2 (100.0) | ND |
| Cefoxitin Screen | 0 (0.0) | 1 (50.0) | ND | ND |
| Clindamycin | 2 (14.3) | 0 (0.0) | 1 (50.0) | 0 (0.0) |
| Erythromycin* | 3 (21.4) | 0 (0.0) | 1 (50.0) | 0 (0.0) |
| Gentamicin* | 0 (0.0) | 0 (0.0) | 0 (0.0) | 0 (0.0) |
| Inducible  Clindamycin* Resistance | 1 (7.1) | 0 (0.0) | 0 (0.0) | 0 (0.0) |
| Levofloxacin* | 0 (0.0) | 0 (0.0) | 0 (0.0) | 0 (0.0) |
| Linezolid* | 0 (0.0) | 0 (0.0) | 0 (0.0) | 0 (0.0) |
| Nitrofurantoin* | 0 (0.0) | 0 (0.0) | ND | ND |
| Oxacillin | 0 (0.0) | 1 (50.0) | ND | 0 (0.0) |
| Rifampicin | 0 (0.0) | 0 (0.0) | ND | 0 (0.0) |
| Teicoplanin* | 0 (0.0) | 1 (50.0) | 1 (50.0) | 0 (0.0) |
| Tetracycline | 2 (14.3) | 1 (50.0) | 1 (50.0) | 1 (100.0) |
| Tigecycline* | 0 (0.0) | 0 (0.0) | 0 (0.0) | 0 (0.0) |
| Tobramycin | 1 (7.1) | 0 (0.0) | 0 (0.0) | 0 (0.0) |
| Trimethoprim-  Sulfamethoxazole | 7 (50.0) | 1 (50.0) | 1 (50.0) | 0 (0.0) |
| Vancomycin* | 0 (0.0) | 0 (0.0) | 1 (50.0) | 0 (0.0) |

Table S1 depicts the number of organisms resistant to antibiotics and their proportions (%) based on the total organisms isolated per species. n = Total number of organisms per species; ND = test for a particular antibiotic was not determined; * Commonly used antibiotics at KNH (25).

***Table S2: Resistance patterns for Gram-negative organisms***

|  | *n (%)* | | | |
| --- | --- | --- | --- | --- |
|  | *Escherichia coli*  *n=13* | *Proteus mirabilis*  *n=9* | *Klebsiella pneumoniae*  *n=6* | *Pseudomonas aeruginosa*  *n=6* |
| Amikacin* | 0 (0.0) | 0 (0.0) | 0 (0.0) | 1 (16.7) |
| Amoxicillin-Clavulanic Acid* | 7 (53.8) | 0 (0.0) | 4 (66.7) | ND |
| Ampicillin* | 13 (100.0) | 6 (66.7) | 5 (83.3) | ND |
| Ampicillin-Sulbactam | 8 (61.5) | 0 (0.0) | 3 (50.0) | ND |
| Aztreonam | 11 (84.6) | 2 (22.2) | 2 (33.3) | ND |
| Cefazolin | 12 (92.3) | 3 (33.3) | 3 (50.0) | 3 (50.0) |
| Cefepime* | 9 (69.2) | 3 (33.3) | 2 (33.3) | 1 (16.7) |
| Cefotaxime | 9 (69.2) | 3 (33.3) | 2 (33.3) | 6 (100.0) |
| Cefoxitin | 4 (30.8) | 0 (0.0) | 0 (0.0) | ND |
| Ceftazidime* | 9 (69.2) | 3 (33.3) | 2 (33.3) | 0 (0.0) |
| Ceftriaxone* | 9 (69.2) | 3 (33.3) | 2 (33.3) | 2 (33.3) |
| Cefuroxime* | 12 (92.3) | 3 (33.3) | 5 (83.3) | 0 (0.0) |
| Cefuroxime Axetil | 9 (69.2) | 2 (22.2) | 2 (33.3) | 1 (16.7) |
| Ciprofloxacin* | 5 (38.5) | 1 (11.1) | 2 (33.3) | 0 (0.0) |
| Gentamicin* | 2 (15.4) | 0 (0.0) | 3 (50.0) | 1 (16.7) |
| Meropenem* | 3 (23.1) | 0 (0.0) | 1 (16.7) | 0 (0.00) |
| Nitrofurantoin* | 0 (0.0) | 3 (33.3) | 1 (16.7) | ND |
| Piperacillin-Tazobactam* | 9 (69.2) | 0 (0. 0) | 2 (33.3) | 1 (16.7) |
| Trimethoprim-Sulfamethoxazole | 10 (76.9) | 5 (55.6) | 4 (66.67) | 0 (0.00) |

Table S2 depicts the number of Gram-negative organisms resistant to antibiotics and their proportions (%) based on the total organisms isolated per species. n = Total number of organisms per species; ND = test for a particular antibiotic was not determined; * Commonly used antibiotics at KNH (25).

***Table S3: Comparison of microbiological and molecular tests for Gram-positive bacteria***

| **Culture-positive for S. aureus & other *Staphylococcus species*** | | **RT-PCR** | |
| --- | --- | --- | --- |
|  |  | *S. aureus* | MRSA |
| *1* | *Staphylococcus aureus* | + | + |
| *2* | *Staphylococcus aureus* | + | + |
| *3* | *Staphylococcus aureus* | + | - |
| *4* | *Staphylococcus aureus* | + | - |
| *5* | *Staphylococcus aureus* | + | - |
| *6* | *Staphylococcus aureus* | - | - |
| *7* | *Staphylococcus aureus* | + | + |
| *8* | *Staphylococcus aureus* | - | + |
| *9* | *Staphylococcus aureus* | + | - |
| *10* | *Staphylococcus aureus* | + | + |
| *11* | *Staphylococcus aureus* | + | - |
| *12* | *Staphylococcus epidermidis* | - | - |
| *13* | *Staphylococcus intermedius* | + | + |
| *14* | *Staphylococcus lentus* | + | + |
| *15* | *Staphylococcus pseudintermedius* | + | - |
| *16* | *Staphylococcus simulans* | - | - |
| *17* | *Staphylococcus xylosus* | - | - |
| *18* | *Suspected contaminants isolated* | + | + |

Table S3 displays RT-PCR results for *S. aureus* and MRSA DNA compared with positive culture results for Gram-positive bacteria. + Positive -Negative

***Table S4: Comparison of microbiological and molecular tests for Gram-negative bacteria***

| **Culture-negative for *S. aureus* & other *Staphylococcus species*** | | RT-PCR | |
| --- | --- | --- | --- |
|  |  | *S. aureus* | MRSA |
| *1* | *Acinetobacter baumannii* | - | - |
| *2* | *Acinetobacter baumannii* | - | - |
| *3* | *Citrobacter freundii* | + | + |
| *4* | *Enterobacter aerogenes* | - | - |
| *5* | *Escherichia coli* | - | - |
| *6* | *Escherichia coli* | - | - |
| *7* | *Escherichia coli* | - | - |
| *8* | *Escherichia coli* | + | - |
| *9* | *Escherichia coli* | - | + |
| *10* | *Escherichia coli* | - | - |
| *11* | *Escherichia coli* | + | + |
| *12* | *Escherichia coli* | - | - |
| *13* | *Klebsiella oxytoca* | - | + |
| *14* | *Klebsiella pneumoniae* | - | - |
| *15* | *Klebsiella pneumoniae* | - | - |
| *16* | *Klebsiella pneumoniae* | - | - |
| *17* | *Pantoea agglomerans* | - | - |
| *18* | *Proteus mirabilis* | - | - |
| *19* | *Proteus mirabilis* | - | - |
| *20* | *Proteus mirabilis* | - | - |
| *21* | *Proteus mirabilis* | - | - |
| *22* | *Proteus mirabilis* | - | - |
| *23* | *Providencia stuartii* | - | - |
| *24* | *Pseudomonas aeruginosa* | + | - |
| *25* | *Pseudomonas aeruginosa* | - | - |
| *26* | *Pseudomonas aeruginosa* | - | - |
| *27* | *Pseudomonas aeruginosa* | - | - |
| *28* | *Raoultella ornithinolytica* | - | + |
| *29* | *Serratia fonticola* | - | - |
| *30* | *Serratia fonticola* | - | - |
| *31* | *Serratia fonticola* | - | - |
| *32* | *Serratia marcescens* | - | + |

Table S4 displays RT-PCR results for *S. aureus* and MRSA DNA compared with positive culture results for Gram-negative bacteria. + Positive -Negative

***Table S5: Distribution of organisms based on culture and RT-PCR results for S. aureus and MRSA***

| n (%) | | | | |
| --- | --- | --- | --- | --- |
|  | Gram-positive | Gram-negative | No growth | **Total** |
| *S. aureus* Detection |  |  |  |  |
| Culture-positive | 14 (56.0) | 0 (0.0) | 0 (0.0) | **14 (16.5)** |
| Culture-negative | 11 (44.0) | 55 (100.0) | 5 (100.00 | **71 (83.5)** |
| RT-PCR-positive | 13 (72.2) | 4 (12.5) | 1 (100.0) | **18 (35.3)** |
| RT-PCR-negative | 5 (27.8) | 28 (87.5) | 0(0.0) | **33 (64.7)** |
|  |  |  |  |  |
| MRSA Detection | |  |  |  |
| Culture-positive | 0 (0.0) | 0 (0.0) | 0 (0.0) | **0 (0.0)** |
| Culture-negative | 25 (100.0) | 55 (100.0) | 5 (100.0) | **85 (100.0)** |
| PCR-positive | 8 (44.4) | 6 (18.8) | 0 (0.0) | **14 (27.5)** |
| PCR-negative | 10 (55.6) | 26 981.3) | 1 (100.0) | **37 (72.5)** |

As shown in Table S5, there were 80 culture-positive and 5 culture-negative tests conducted whereas only 51 RT-PCR tests took place
